# Hourly values of an advanced human-biometeorological index for diverse populations from 1991 to 2020 in Greece

**DOI:** 10.1038/s41597-024-02923-y

**Published:** 2024-01-16

**Authors:** Christos Giannaros, Ilias Agathangelidis, Elissavet Galanaki, Constantinos Cartalis, Vassiliki Kotroni, Konstantinos Lagouvardos, Theodore M. Giannaros, Andreas Matzarakis

**Affiliations:** 1 National and Kapodistrian University of Athens, Department of Physics, 15784 Athens, Greece; 2 National Observatory of Athens, Institute for Environmental Research and Sustainable Development, Palea Penteli, 15236 Athens, Greece; 3 German Meteorological Service (DWD), Research Centre Human Biometeorology, D-79085 Freiburg, Germany; 4 University of Freiburg, Institute of Earth and Environmental Sciences, D-79104 Freiburg, Germany

**Keywords:** Climate sciences, Environmental sciences

## Abstract

Existing assessments of the thermal-related impact of the environment on humans are often limited by the use of data that are not representative of the population exposure and/or not consider a human centred approach. Here, we combine high resolution regional retrospective analysis (reanalysis), population data and human energy balance modelling, in order to produce a human thermal bioclimate dataset capable of addressing the above limitations. The dataset consists of hourly, population-weighted values of an advanced human-biometeorological index, namely the modified physiologically equivalent temperature (mPET), at fine-scale administrative level and for 10 different population groups. It also includes the main environmental drivers of mPET at the same spatiotemporal resolution, covering the period from 1991 to 2020. The study area is Greece, but the provided code allows for the ease replication of the dataset in countries included in the domains of the climate reanalysis and population data, which focus over Europe. Thus, the presented data and code can be exploited for human-biometeorological and environmental epidemiological studies in the European continent.

## Background & Summary

Extreme environmental conditions related to heat and cold pose a major threat to human health, well-being, performance and productivity^1^. Heat waves in particular, cause more premature deaths than any other weather-related natural disaster^2^, especially in cities due to the urban overheating^3,4^. Given the increasing risk of deadly atmospheric conditions due to the anthropogenic climate change^5,6^, there is an imperative need for improved heat-health management and action planning^7^. In this direction, assessing comprehensively the thermal environment and its impact on human life is necessary. This assessment should be based on three main pillars^1,8–18^:

1. Analysis of the combined effects of the objective (environmental) and subjective (physiological) parameters associated with the human thermal sensation using rational indices (i.e. indices based on the human energy balance equation).
2. Use of data at spatiotemporal scales that are relevant to the population exposure and epidemiological outcomes.
3. Consideration of diverse populations, including the most vulnerable, through the simulation of variable physiological settings.

The existing heat- and health-related literature is subject to one or more limitations with respect to the above requirements. Most studies, especially in the field of environmental epidemiology, have used primarily air temperature to characterize thermal exposure^6,19–21^. The application of this oversimplified approach is mainly driven by the availability of thermal-related meteorological data, which are traditionally based on surface weather stations^11,13,19,22,23^. Direct indices, such as the apparent temperature^24^ and wet-bulb globe temperature^25^, have also been used^26–28^ in an effort to appraise the human thermal bioclimate based on the collective impact of its main environmental drivers (air temperature, vapour pressure (humidity), wind speed, and short- and long-wave radiation fluxes). Still, the physiological basis of the human thermal environment, as expressed by demographical and personal characteristics, metabolism and clothing, is neglected^8–12^, while the ground-based data used are usually unrepresentative of the population exposure due to the location (e.g. airports) of the meteorological stations from which they are mainly derived^11,13,19,22,23^ and/or their sparse spatiotemporal availability.

Aiming at addressing the above issues, especially in regions characterized by limited spatial coverage of surface observational networks, recent studies exploited climate retrospective analysis (reanalysis) datasets^13–15,23,29–36^. The wealth of data provided by reanalyses allows for retrieving simple metrics related to the thermal environment or even computing rational indices, such as the physiological equivalent temperature (PET)^37,38^ and universal thermal climate index (UTCI)^39,40^. The gridded structure of reanalyses also allows for estimating thermal sensation at population-weighted spatial scales, thus better representing the human thermal bioclimate. This is achieved by weighting the average of the variables of interest (e.g. air temperature) towards the most densely populated grid cells within the study area. This approach is particularly useful in regions characterized by large topographic and population heterogeneity^13–15^. Currently, the ERA5^41^ and ERA5-Land^42^ datasets at ~31 and ~9 km spatial resolution, respectively, represent the state-of-the-art in global reanalysis, providing high quality data for human-biometeorological and environmental epidemiological applications. Yet, they are prone to misrepresentation errors in areas characterized by complex topography and/or proximity to the sea^14,34,43–45^, mostly due to their coarse horizontal grid resolution (ERA5) and grid coverage (ERA5-Land), which is determined by a land-sea mask with values greater than 50%^46^.

Concerning the third human-biometeorological analysis pillar mentioned above, the physiological characteristics of different subjects and/or population groups have been rarely considered^47–49^. This diminishes the value of the human physiological thermal responses, which are critical for characterizing and dealing with vulnerability^50^, and it is an outcome of the simplification arising from using standard meteorological variables or simple composite indices. Even the increasing exploitation of UTCI is based on a six-order polynomial approximation of the thermo-physiological version of the index (UTCI-Fiala)^40^, derived from a regression analysis that conducted for a fixed physiological setting and a maximum exposure time of 2 h^15,23,30,33,34,51^. Thus, the polynomial-based UTCI estimates cannot account for diverse populations^18,52^. The same also applies for PET, as constant values for clothing and activity are primarily considered for its computation^12,18,37,53^.

**Fig. 1:**
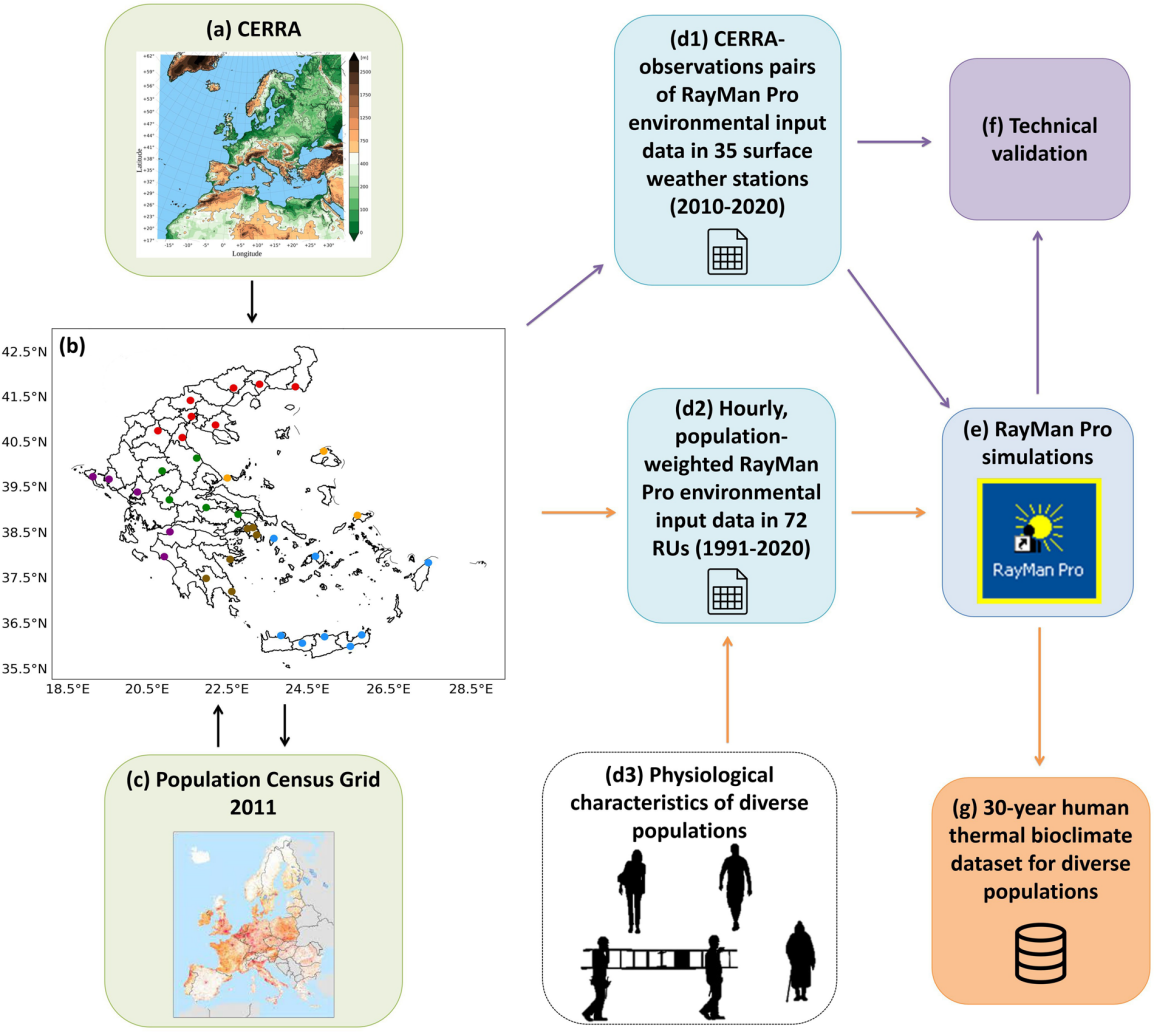
Data generation workflow, including (**a**) the CERRA domain (adopted from El-Said *et al*.^64^), and (**b**) map of Greece (Lambert Conformal Conic projection) with identification of the 72 RUs (black boundaries) and the 35 surface weather stations operated by the NOA Meteo Unit^63^ in six different geographical regions (eight red dots in North Greece, five green dots in Central Greece, five purple dots in West Greece, three orange dots in North Aegean, six brown dots in South Greece and eight blue dots in South Aegean).

The present work, which is conducted in the framework of the HEAT-ALARM research project, aims at assisting the human-biometeorological and environmental epidemiological communities to overcome the above limitations. For this, it provides a 30-year (1991–2020) dataset of population-weighted modified PET (mPET)^54,55^, computed for 10 population subsets through the RayMan Pro model^56–60^ in 72 regional units and combinations thereof (RUs), which are based on the NUTS-3 (Nomenclature of Territorial Units for Statistics-3) classification in Greece, using the Copernicus European Regional Reanalysis (CERRA)^61^ at 5.5 km spatial resolution (Fig. 1a–c,d2,d3,e,g). It also provides the code for replicating the dataset, not only directly in Greece, but also in any other country included in the CERRA domain after appropriate adjustments. It is worth noting that the provided data, which include the main mPET environmental drivers, have a temporal resolution of 1 h, as exposure duration is a key factor to heat and cold stress health-related outcomes^50,52,62^. Further, a comprehensive validation of mPET and its main environmental drivers accompanies the dataset (Fig. 1a,b,d1,e,f). The validation in based on up to 11-year observations, including surface solar radiation downwards, obtained from 35 ground-based weather stations of the nationwide network operated by the METEO Unit at the National Observatory of Athens (NOA)^63^. This is of great importance, as existing datasets of thermal indices have not extensively validated, mainly due to the lack of high-quality ground-truth data^23^.

## Methods

### CERRA data

The CERRA dataset constitutes the most recent regional climate reanalysis released by the Copernicus Climate Change Service (C3S)^61^. Its production is based on the HARMONIE-ALADIN numerical weather prediction (NWP) and data assimilation (DA) system. DA is performed under a 3-h cycling mode using most observations that are available through a three-dimensional variational (3D-var) assimilation scheme for the upper-level atmospheric data and an optimal interpolation (OI) technique for the surface analysis. The ERA5 reanalysis is also used during the NWP initialization with DA for providing the lateral boundary conditions to the model. 30 h forecasts are produced at the 0000 and 1200 UTC (universal time coordinated) assimilation cycles, while 6 h forecasts are provided at the rest of the assimilation times. The domain of CERRA focuses over Europe (Fig. 1a) with a horizontal and vertical resolution of 5.5 km and 106 levels, respectively^61,64,65^. The high spatial resolution of CERRA that allows for a better description of topographic and physiographic features, combined with the increased number of observations assimilated in the system provides a significant added value to the dataset compared to the current state-of-the-art global reanalyses.

The first step towards the realization/replication of the presented human thermal bioclimate dataset involves the acquisition of the CERRA data from the C3S Climate Data Store (CDS)^61^. The data are downloaded in GRIB format, covering the whole CERRA domain. Then, a regional subset of the original data is created in order to reduce the processing time in the next implementation steps. The subset focuses over Greece (19 °E to 30 °E and 34.5 °N to 42 °N) in the present paper (Fig. 1b), but the regional boundaries can be tailored to the user needs, based on the provided code, for applications in other countries included in the CERRA domain. The same also applies for the data period, which spans from 1991 to 2020 in the current paper. The derived data include: (a) 2-m air temperature, (b) 2-m relative humidity, (c) 10-m wind speed, (d) surface solar radiation downwards, (d) skin temperature, (e) albedo, (f) surface roughness, (g) orography and (h) land-sea mask. The latter two variables are retrieved based on a single model time-step, since they represent static geo-information. The rest of the data are obtained at hourly intervals based on the first 3 h forecasts of each assimilation cycle. Instantaneous values correspond to each hourly data record, except from the surface solar radiation downwards, which is an accumulated parameter given in J/m^2 ^^61^. Thus, conversion of this variable in hourly values in W/m^2^ is performed following the C3S guidelines^66^.

### Population weighting

**Fig. 2:**
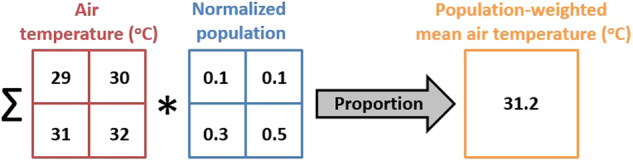
Example of population weighting for a hypothetical regional unit that contains four CERRA grid cells. Each grid cell is indicatively characterized by an hourly temperature estimate and a normalized population value (e.g. 50% of population resides in the bottom right grid cell). Using Eq. (1), the RU temperature mean is weighted by population, providing thus a closer approximation of the temperature experienced by the RU population (31.2 °C).

The population data used in the present paper were retrieved from the GEOSTAT 2011 grid dataset, version 2.0.1^67^. This dataset is the result of the homonymous initiative, which was jointly taken by the Statistical Authority of the European Union (Eurostat) and the National Statistical Institutes (NSIs) of Europe. It contains population grid information at 1 km spatial resolution, based on the 2011 Census data provided by NSIs (Fig. 1c). The first step in the population data processing procedure involves the extraction of the relevant data for Greece by sub-setting the tabular format population data using the identification code for the country. Subsequently, this subset is merged with the GEOSTAT spatial reference grid (European Terrestrial Reference System 1989 Lambert Azimuthal Equal Area projection) using the corresponding grid cell codes. Next, the GEOSTAT grid points are grouped based on the RUs they are contained in or intersect with. The population value of each GEOSTAT grid point (number of inhabitants) is then normalized by dividing it with the sum of the population of each corresponding RU. Then, the GEOSTAT grid is re-projected to the CERRA coordinate system (Lambert Conformal Conic projection) using nearest neighbours, and the sum of the normalized GEOSTAT population grid point included in each CERRA grid cell is computed. Only CERRA cells with land mask values greater than zero are used in this processing step. Using the normalized population sum per CERRA grid cell, the population-weighted centroid per RU is next computed. Finally, the CERRA grid points and population data within each RU are combined in order to compute hourly population-weighted means of the derived reanalysis data, using the following formula:

1 $$\bar{V}={\sum }_{i=1}^{N}{{V}_{i}\ast P}_{i}$$

where $\bar{V}$ is the population-weighted mean of the variable of interest, i spans from 1 to the total number (N) of CERRA grid cells that are contained in each RU, V_i_ is the value of the variable of interest in the i^th^ grid cell and P_i_ is the normalized population resided within the i^th^ grid cell. Figure 2 illustrates an example application of the above population weighting technique.

### mPET computation

PET was first introduced in 1987 by Mayer and Höppe^68^ and its computation is based on the Munich Energy-balance Model for Individuals (MEMI), which simulates the human thermoregulation system according to a two-node body model and a single-layer clothing model, assuming primarily constant levels of both metabolic rate and clothing insulation^12,18,37^. mPET, the modified version of PET, has been recently developed in order to address PET limitations associated with the influence of vapour pressure and clothing insulation on the heat exchange processes over the human body. The improved performance of mPET with respect to air humidity and clothing factors is achieved through the adoption of an enhanced semi-steady human energy balance model. Compared to MEMI, the mPET thermoregulation model combines a multiple-node body model (15–25 nodes) with a multiple-layer clothing model (1–3 layers). This allows for a more realistic simulation of the vasoconstriction and vasodilation phenomena, the blood and body heat transfer, and the clothing insulation and vapour resistance effectiveness^54,55^. It also allows for clothing and activity modifications^12,18^, which are necessary when considering the sensitivities of diverse populations to thermal stress. Thus, mPET constitutes a universally applicable human-biometeorological index under different climatic contexts and various research and operational activities^49,69–71^.

The computation of mPET in the present paper is performed with the use of the RayMan Pro model, version 3.1^56^–^60^, on the basis of calculating the mean radiant temperature, which is an important input variable for the simulation of rational thermal indices, as it parameterizes the impact of the short- and long-wave radiation fluxes on the human energy balance. Its estimation in the RayMan Pro model is based on the provided surface solar radiation downwards^56,57^. Other environmental input data include the 2-m air temperature, 2-m relative humidity, skin temperature, albedo and 1.1-m wind speed, computed according to the 10-m wind speed and surface roughness^72^, in order to approximate the weighting height of the human body^71^. The latter height builds the reference level for the computation of mPET. All data are imported in plain text format into the RayMan Pro model as hourly population-weighted means at the locations of the population-weighted centroids of the 72 defined regional units in Greece, based on the data processing described in the previous sub-sections. Concerning the physiological parameters provided to the mPET thermoregulation model within RayMan Pro, they include anthropometric data (sex, age, height and weight) and activity metabolic rate for 10 population groups, as follows:

1. Female Adult: 35 years old, weighting 65 kg and having a height of 1.65 m, and performing low physical activity (80 W).
2. Male Adult: 35 years old, weighting 75 kg and having a height of 1.75 m, and performing low physical activity (80 W).
3. Female Senior: 70 years old, weighting 60 kg and having a height of 1.65 m, and being at rest (50 W).
4. Male Senior: 70 years old, weighting 70 kg and having a height of 1.75 m, and being at rest (50 W).
5. Female Worker 1: 35 years old, weighting 65 kg and having a height of 1.65 m, and performing light labour (110 W).
6. Male Worker 1: 35 years old, weighting 75 kg and having a height of 1.75 m, and performing light labour (110 W).
7. Female Worker 2: 35 years old, weighting 65 kg and having a height of 1.65 m, and performing moderate labour (210 W).
8. Male Worker 2: 35 years old, weighting 75 kg and having a height of 1.75 m, and performing moderate labour (210 W).
9. Female Worker 3: 35 years old, weighting 65 kg and having a height of 1.65 m, and performing heavy labour (310 W).
10. Male Worker 3: 35 years old, weighting 75 kg and having a height of 1.75 m, and performing heavy labour (310 W).

For all population subsets, the clothing insulation is automatically adjusted based on the thermal conditions provided to the model^12,54,55^. The choice of these particular populations aims at permitting the inclusion of sex equity and increased vulnerability of elderly and outdoor workers in human-biometeorological and heat-health-related assessments. Especially the latter group of people is often overlooked in heat prevention planning despite the increasing heat-related occupational injuries and fatalities, and the fact that physiological strain in workplaces leads to significant labour productivity losses^73–75^. The inclusion of three different profiles of outdoor workers reflects the variability in the intensity of the work needed in the major outdoor occupation sectors (e.g. agriculture)^76^. It should be noted that other population subsets can also been considered, as the above physiological characteristics can been fine-tuned in the RayMan Pro model according to the user needs through the provided code. The final output of the model is provided in a text file, and it includes the computed mPET values, as well as various information concerning date, time, location and input data.

## Data Records

The presented human thermal bioclimate dataset is publicly available at the Zenodo repository^77^. It contains an Excel file that includes the RUs metadata, namely the ID, name and included municipalities for each one of the 72 regional units and combinations thereof, defined based on the NUTS-3 classification in Greece. It also contains a set of 72 comma-separated values (csv) files, created by processing the original RayMan Pro output files for each RU. Each csv is named after the RUs ID (e.g. “RU_1_human_bioclimate.csv”) and it includes the hourly values of population-weighted mPET, simulated for the period 1991–2020 and for the 10 groups of people described in the previous section. The structure of each csv file is as follows:

- The first four columns contain date (year, month, day) and time (0–23 in UTC format) information.
- The next five columns include the main environmental drivers and modifiers of mPET, namely the 2-m air temperature (T2), 2-m relative humidity (RH2), 1.1-m wind speed (WS1.1), skin temperature (TSK) and surface solar radiation downwards (SSRD), provided as population-weighted input into the RayMan Pro model.
- The 10th column contains the population-weighted mean radiant temperature (Tmrt) values computed by the RayMan Pro model.
- The final 10 columns provide the values of population-weighted mPET, simulated by the RayMan Pro model for the 10 targeted population subsets, namely female adult (mPET_f_adult), male adult (mPET_m_adult), female senior (mPET_f_senior), male senior (mPET_m_senior), female worker 1 (mPET_f_worker_1), male worker 1 (mPET_m_worker_1), female worker 2 (mPET_f_worker_2), male worker 2 (mPET_m_worker_2), female worker 3 (mPET_f_worker_3) and male worker 3 (mPET_m_worker_3).

A README.txt file provides information on the dataset and its structure, as described above.

## Technical Validation

**Fig. 3:**
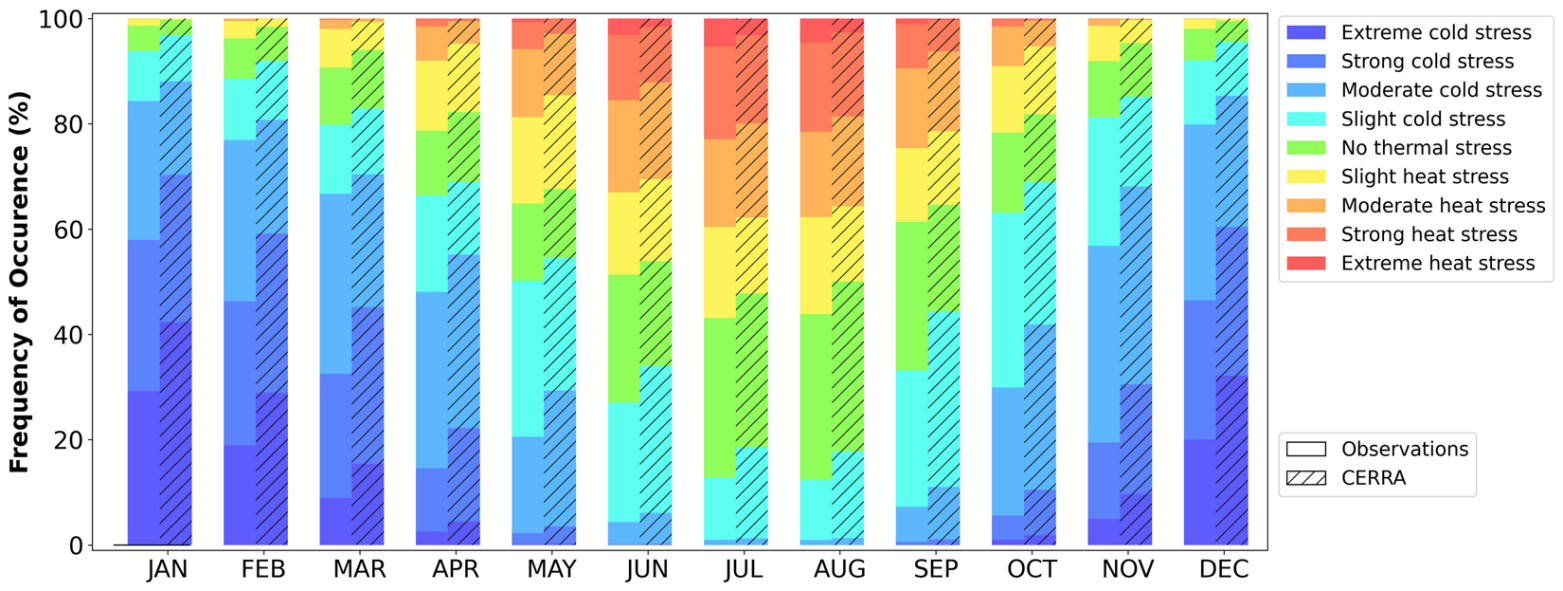
Thermal bioclimate diagram for Greece, presenting the monthly frequencies of occurrences of the mPET thermal stress classes according to the observational and CERRA-based data that cover the period from 2010 to 2020 (mPET thermal stress classification after Matzarakis *et al*.^38^).

mPET and its main environmental drivers are evaluated against observations from 35 surface weather stations (Fig. 1a,b,d1,e,f) operated in Greece by the NOA Meteo Unit for the period 2010–2020^63^. The validation is based on hourly CERRA-observations pairs of 2-m air temperature and relative humidity, 1.1-m wind speed and surface solar radiation downwards, derived according to the “nearest neighbour” technique^78^, and Tmrt and mPET, computed by the RayMan Pro model at the locations of the weather monitoring sites for the “Male Adult” physiological setting (see “Methods” section). Using the data pairs in particular, domain-wide statistics, namely the mean bias (MB), root mean square error (RMSE) and index of agreement (IOA), are used as an overall measure of the capability of CERRA to reproduce the observed human-biometeorological conditions. The geographical and seasonal dependence of the statistics is investigated by considering six geographical regions (Fig. 1b) and four seasons (DJF: December-January-February; MAM: March-April-May; JJA: June-July-August; SON: September-October-November), respectively (Table 1). Further, the observed and modelled relative frequencies of mPET thermal stress classes are compared on a monthly basis through a bioclimate diagram constructed using the data pairs from all stations (Fig. 3).

**Table 1:** CERRA performance statistics for mPET and its environmental drivers computed for regional and seasonal data subsets.

Region	Statistical metric	T2 (°C)	RH2 (%)	WS1.1 (m/s)	SSRD (W/m^2^)	Tmrt (°C)	mPET (°C)
North Greece	MB	−0.3	−3.0	0.7	17.7	0.2	−1.3
	RMSE	1.9	11.3	1.2	105.3	4.9	3.1
	IOA	1.0	0.9	0.7	1.0	1.0	1.0
North Aegean	MB	−1.2	2.1	0.8	14.9	−0.7	−2.0
	RMSE	2.0	10.3	1.4	90.6	4.4	3.2
	IOA	1.0	0.9	0.7	1.0	1.0	1.0
Central Greece	MB	0.4	−3.00	0.85	13.55	0.62	−1.17
	RMSE	1.9	12.4	1.3	109.0	5.3	3.1
	IOA	1.0	0.7	0.7	1.0	1.0	1.0
West Greece	MB	−0.6	−3.17	0.67	1.99	−0.98	−1.9
	RMSE	2.1	12.6	1.1	88.8	4.6	3.3
	IOA	1.0	0.9	0.6	1.0	1.0	1.0
South Greece	MB	−0.7	−2.6	0.5	11.2	−0.6	−1.5
	RMSE	1.8	10.5	1.0	98.6	4.6	2.9
	IOA	1.0	0.9	0.8	1.0	1.0	1.0
South Aegean	MB	−0.4	−0.3	1.1	14.0	0.1	−1.4
	RMSE	1.5	10.3	1.8	97.7	4.4	2.8
	IOA	1.0	0.8	0.7	1.0	1.0	1.0
**Season**	**Statistical metric**	**T2 (°C)**	**RH2 (%)**	**WS1.1 (m/s)**	**SSRD (W/m**^2^**)**	**Tmrt (°C)**	**mPET (°C)**
DFJ	MB	−0.8	−0.2	0.9	9.6	−0.4	−1.9
	RMSE	2.0	10.1	1.5	76.8	4.6	3.1
	IOA	1.0	0.9	0.8	0.90	1.0	0.9
MAM	MB	−0.3	−1.9	0.7	18.4	0.1	−1.4
	RMSE	1.8	11.6	1.3	119.6	5.2	3.2
	IOA	1.0	0.9	0.8	1.0	1.0	1.0
JJA	MB	0.0	−2.8	0.8	16.4	0.3	−1.0
	RMSE	1.8	11.9	1.3	110.6	4.5	2.9
	IOA	1.0	0.9	0.8	1.0	1.0	1.0
SON	MB	−0.5	−2.2	0.8	8.6	−0.3	−1.6
	RMSE	1.8	11.1	1.3	87.0	4.5	3.0
	IOA	1.0	0.9	0.8	1.0	1.0	1.0

As shown in Table 1, CERRA primarily simulate cooler and drier near-surface conditions compared to the observed ones throughout the year (MBs <0), with RMSEs ranging from 1.5 °C and 10.3% in South Aegean to 2.1 °C and 12.6% in West Greece. For WS1.1, the MB values indicate CERRA overestimation, with the greatest RMSEs being recorded in the North (1.4 m/s) and South (1.8 m/s) Aegean. T2 and WS1.1 are better modelled during the warm period of the year (MAM and JJA), whereas the lowest RH2 deviations from the observations are found during the DJF season (10.1%). CERRA SSRD biases are positive, with their dependence being mainly seasonal, as significantly higher RMSEs are evident during MAM (119.6 W/m^2^) and JJA (110.6 W/m^2^). Given that Tmrt is highly dependent on radiation fluxes^56^,^57^, the SSRD biases are partially reflected in this thermal-related variable. Thus, the greatest Tmrt errors are found in the course of MAM (5.2 °C) and in Central Greece (5.3 °C), which are positive in terms of MB. In the rest of the regions and seasons examined, the Tmrt deviations from observations are lower than 5 °C, with mixed results with respect to model overestimation/underestimation. Concerning mPET, its observational-based values are underestimated by CERRA (MBs < 0) with RMSEs being lower than 3.5 °C. Overall, CERRA performs better at reproducing the observed mPET during the summer (JJA) and over the South continental and insular Greece. The IOA values, which represent the correlation between the modelled and observed data, are mostly greater than 0.7 across all regions and for all seasons and variables (higher than 0.9 specifically for Tmrt and mPET). This outcome denotes that CERRA can reliably simulate the observed diurnal variation of the examined human thermal bioclimate parameters (Table 1). It should be noted that the values of the above statistical metrics are comparable to reanalyses inaccuracies reported in previous studies^44,79,80^. Particularly for Tmrt, the presented RMSEs are significantly lower compared to those provided by Di Napoli *et al*. (8.6 ± 2.5 °C)^33^ and Yan *et al*., (9.5 °C with a range of 7.1 °C to 12.1 °C)^34^, while the CERRA mPET statistics are similar to (or even better than) those reported in regional modelling studies^78,81^. Moreover, Galanaki *et al*.^46^ demonstrated the quality of CERRA in comparison to ERA5-Land for human-biometeorological applications, especially in the course of the summer and in complex geomorphological regions in Greece, using the same observational dataset as in the current technical validation. It is worth noting that more than 10 stations in this dataset are located in urban areas. Thus, the CERRA validation outcomes are relevant to urban environments, which are usually the most densely populated areas. This is of great importance, especially in relation to the population weighting technique applied to the raw CERRA data (see “Methods” section). Nonetheless, it should be clarified that urban-scale land and atmospheric processes and features are not parameterized in the modelling systems used for producing CERRA, as well as ERA5-Land and other reanalysis datasets. This is a notabe shortcoming, as not properly simulating the urban effects can lead to degraded reanalysis accuracy in built-up areas^80^. The incorporation of advanced techniques and tools, such as improving urban land cover representation^82^ and/or coupling with urban canopy models^83,84^, even though computationally expensive, can provide a more accurate representation of the urban environments, leading to improved climate-relevant data and assessments in cities.

The overall good performance of CERRA in simulating the human thermal bioclimate in Greece is further demonstrated in Fig. 3. The differences between the observed and modelled frequencies of occurrence for the mPET classes from slight to extreme heat stress are below 5% throughout the year. These differences are even lower (<2.5%) during the warm months (April to October) for the strong and extreme heat stress classes. This is of great importance, as it highlights the applicability of CERRA for heat stress related human-biometeorological and environmental epidemiological studies. The quality of the CERRA performance degrades during January and December, as greater than 10% deviations are found for the mPET class of extreme cold. Further, moderate CERRA-observations differences (5–10%) are evident for the slight cold stress class during the warm period and for the strong and moderate cold stress classes during the cold period, while the observed mPET values corresponding to no thermal stress are generally well simulated by CERRA in the course of all months (Fig. 3).

## Usage Notes

### Data reconstruction/replication

The code for replicating the presented human thermal bioclimate dataset includes: (i) a plain text document providing commands for installing Python, version 3.11, and relevant packages in a Conda environment, (ii) 10 executable scripts in Python, Bash and Visual Basic (VB) programming languages, (iii) two supporting files for building the Conda environment and defining the CERRA coordinate reference system, (iv) the “GEOSTAT” folder, and the (v) the “regional_units” folder, which includes the defined regional units in Greece in GeoPackage format. In addition to the above, the user should download the compressed folder (GEOSTAT-grid-POP-1K-2011-V2-0-1.zip) that contains the GEOSTAT 2021 grid dataset, version 2.0.1^67^, and unzip it within the “GEOSTAT” folder. The terms of use of the GEOSTAT 2011 data can be found in the document GEOSTAT_grid_POP_1K_2011_V2_0_1_UsageConditions.pdf within GEOSTAT-grid-POP-1K-2011-V2-0-1.zip^67^. Further, the user should request the compressed folder (RayManPro.zip), which contains the RayMan Pro, version 3.1, model^56–60^, from the website of Prof. Dr. Matzarakis (https://www.urbanclimate.net/rayman/introraymanpro.htm). RayManPro.zip should be then stored and unzipped within the main code directory. The RayMan Pro model^56–60^ is free for use for academic and scientific purposes.

The commands related to setting up the Conda environment can be executed as a whole or in part. They can also be easily adapted to Windows and other platforms. The executable code can be directly executed under the indicated priority order for reproducing the full dataset (1991–2020) in the defined RUs in Greece (Fig. 1a–c,d2,d3,e,g). For replicating the dataset in another country included in the CERRA domain, the user should provide the desired regional units in the same format. He/she also needs to adapt the script for creating the regional subset of the original CERRA data, and the scripts related to the population-weighted processing. Detailed instructions on these modifications are provided with comments within the scripts. Overall, the provided code and documentation allows for various adaptations according to the user needs.

### Data and code use cases

The provided/replicated data can be widely used in human-biometeorological and environmental epidemiological research. Potential applications at fine-scale administrative level and 1 h temporal resolution include:

- Assessment of the human thermal bioclimate and its drivers in the recent past (1991–2020)^29,31^ for supporting applications in various sectors, such as in tourism and recreational health^85^.
- Long-term analysis of population exposure to heat and cold stress extremes by combining the dataset with population data^86,87^.
- Examination of the exposure-response relationship between thermo-physiological thermal stress and mortality by combining the dataset with death counts^13,23,36^.

It is important to note that all use cases exploiting the data can consider the differences in the human thermoregulation responses among diverse populations in order to better understand, assess and cope with thermal stress vulnerability.

Further, the code includes scripts that can be used as stand-alone, including those for deriving population-weighted variables from gridded climate datasets, and the VB script for automatically launching RayMan Pro simulations in order to compute rational human thermal indices, and especially mPET for various population subsets.

## Data Availability

The code used to produce the presented human thermal bioclimate dataset is publicly available at the Zenodo repository^88^. It is free to re-use and modify with attribution under the Creative Commons Attribution 4.0 International (CC BY 4.0) license.
